# Supplementary figures and images for: Physiological and Transcriptomic Mechanisms Underlying Vitamin C-Mediated Cold Stress Tolerance in Grafted Cucumber
Source: Plants (Basel). 2025 Aug 2;14(15):2398. doi: 10.3390/plants14152398 (PMC12349555; doi:10.3390/plants14152398)

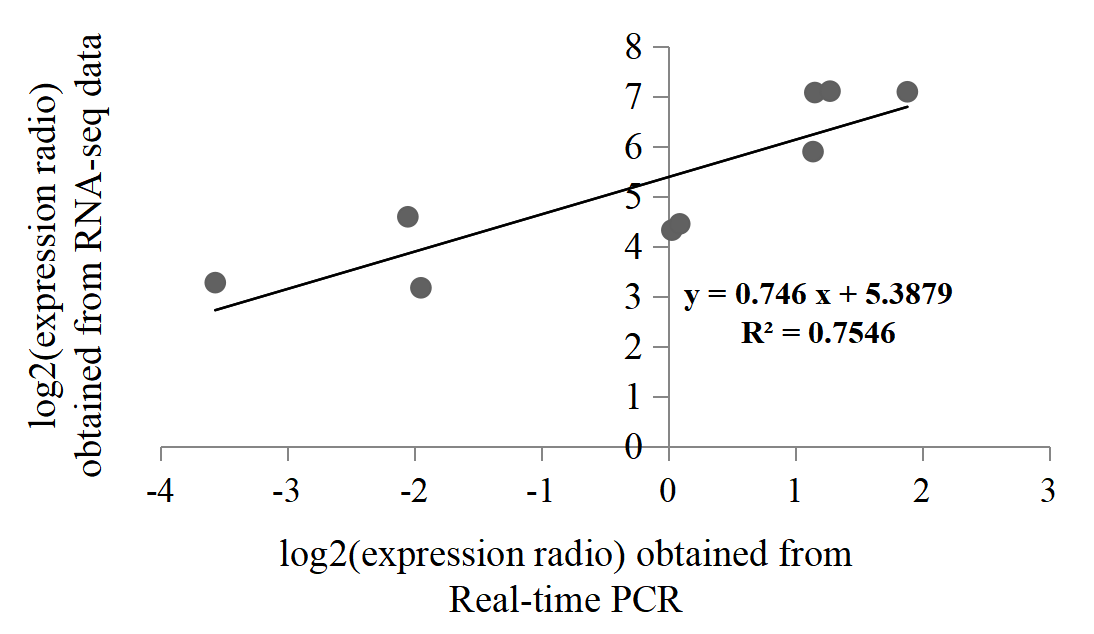

Supplement: Supplementary file 1 [file plants-14-02398-s001.zip › Figure S1.tif]
